# How Math Anxiety Relates to Number–Space Associations

**DOI:** 10.3389/fpsyg.2016.01401

**Published:** 2016-09-14

**Authors:** Carrie Georges, Danielle Hoffmann, Christine Schiltz

**Affiliations:** ^1^Institute of Cognitive Science and Assessment, Research Unit Education, Culture, Cognition and Society, Faculty of Language and Literature, Humanities, Arts and Education, University of LuxembourgEsch-Belval, Luxembourg; ^2^Luxembourg Centre for Educational Testing, Faculty of Language and Literature, Humanities, Arts and Education, University of LuxembourgEsch-Belval, Luxembourg

**Keywords:** math anxiety, basic number skills, number–space associations, SNARC effect, working memory

## Abstract

Given the considerable prevalence of math anxiety, it is important to identify the factors contributing to it in order to improve mathematical learning. Research on math anxiety typically focusses on the effects of more complex arithmetic skills. Recent evidence, however, suggests that deficits in basic numerical processing and spatial skills also constitute potential risk factors of math anxiety. Given these observations, we determined whether math anxiety also depends on the quality of spatial-numerical associations. Behavioral evidence for a tight link between numerical and spatial representations is given by the SNARC (spatial-numerical association of response codes) effect, characterized by faster left-/right-sided responses for small/large digits respectively in binary classification tasks. We compared the strength of the SNARC effect between high and low math anxious individuals using the classical parity judgment task in addition to evaluating their spatial skills, arithmetic performance, working memory and inhibitory control. Greater math anxiety was significantly associated with stronger spatio-numerical interactions. This finding adds to the recent evidence supporting a link between math anxiety and basic numerical abilities and strengthens the idea that certain characteristics of low-level number processing such as stronger number–space associations constitute a potential risk factor of math anxiety.

## Introduction

Math anxiety has been defined as an emotional response evoked in some individuals when dealing with numbers and mathematical problems, ultimately disrupting their performance (Suárez-Pellicioni et al., 2015). The prevalence of math anxiety is much higher than previously assumed with more than 30% of 15-year old students from “Organization for Economic Co-operation and Development” countries reporting feelings of tension or nervousness when solving math problems in school or at home (OECD, 2013). Considering the importance of mathematics in our highly technological society and thus the potentially far-reaching consequences of math anxiety, it is crucial to get a better understanding of the factors contributing to it to facilitate early identification, prevention, and remediation.

Although, it remains largely unclear how math anxiety actually develops, it is generally assumed to have multiple origins, with both social influences and cognitive predispositions playing a role in its development. Moreover, an association between math anxiety and gender is commonly reported, with women featuring greater math anxiety than men throughout their entire schooling period (Hembree, 1990; Devine et al., 2012).

The most commonly studied cognitive variables associated with math anxiety are without a doubt arithmetic performance and working memory (WM; e.g., Ashcraft and Faust, 1994; Ashcraft and Kirk, 2001; Ashcraft et al., 2007; Passolunghi et al., 2016). Recent evidence, however, suggests that math anxiety not only relates to performance deficits on complex arithmetic tasks, but also concerns ***basic numerical processing*** (Maloney et al., 2010, 2011; Núñez-Peña and Suárez-Pellicioni, 2014; Dietrich et al., 2015). For instance, individuals with high math anxiety (HMA) were shown to differ from their low math anxious (LMA) peers on tasks as simple as enumerating items in the counting range (Maloney et al., 2010). Moreover, HMA individuals displayed stronger numerical distance effects (NDEs) in both behavioral (Maloney et al., 2011; Dietrich et al., 2015) and ERP settings (Núñez-Peña and Suárez-Pellicioni, 2014). Maloney et al. (2011) considered these findings as evidence for a less precise numerical magnitude representation, i.e., a deficit in the approximate number system (ANS), in HMA individuals. Since Dietrich et al. (2015) did, however, not find a relation between math anxiety and the NDE when using a non-symbolic dot comparison task (i.e., the standard task to measure ANS acuity; De Smedt et al., 2013), but only with performance in symbolic number comparison, they suggested that impairment of the latter comparison processes rather than a less precise ANS might constitute a risk factor for the development of math anxiety. In addition to this, Young et al. (2012) reported that children with HMA showed reduced activity in brain regions known to support numerical processing, such as the dorsolateral prefrontal cortex and posterior parietal lobe, during an addition and subtraction verification task. Moreover, Rubinsten and Tannock (2010) observed a strong relationship between developmental dyscalculia and math anxiety. Altogether, these findings thus suggest that basic numerical deficits likely contribute to the emergence of math anxiety, possibly via compromising the development of high-level mathematical skills (Holloway and Ansari, 2009).

Math anxiety has also been negatively associated with basic non-numerical abilities such as ***spatial skills*** (Maloney et al., 2012; Ferguson et al., 2015), suggesting that the deficits observed in HMA individuals extend beyond numerical activities proper. For instance, Maloney et al. (2012) found a strong negative correlation between math anxiety and the spatial visualization scale of the Object Spatial Imagery Questionnaire (Blajenkova et al., 2006), comprising no math-related content. Moreover, individuals with HMA performed worse than their LMA peers on the paper-and-pencil mental rotation test (MRT; Maloney, 2011). This observation could be replicated by Ferguson et al. (2015) using a different measure. A possible explanation for these findings is that poor spatial abilities prevent optimal math achievement (e.g., Gunderson et al., 2012), thereby leading to the development of math anxiety.

Considering the relationships between math anxiety and deficits in basic numerical (Maloney et al., 2011; Núñez-Peña and Suárez-Pellicioni, 2014; Dietrich et al., 2015) and small-scale spatial skills (Maloney et al., 2012; Ferguson et al., 2015) as well as the recently proposed idea that these factors might be at the origin of math anxiety, the present study aimed to determine whether the quality of spatial-numerical associations might also be a potential risk factor of math anxiety.

During binary classification judgments on single Arabic digits, individuals usually tend to be faster for small/large numbers with their left/right hand respectively. This phenomenon, known as the SNARC (spatial-numerical association of response codes) effect, is considered as behavioral evidence for a tight relationship between numerical and spatial representations, with small/large digits being associated with the left/right side of space respectively (Dehaene et al., 1993). Despite the fact that the SNARC effect has been extensively replicated, its cognitive origin remains debated. The dominant and most traditional visuospatial account is based on the idea that numbers are mentally represented along a continuous left-to-right-oriented representational medium (the mental number line) with small/large numbers located on the left/right side of the continuum respectively (Moyer and Landauer, 1967; Restle, 1970; Dehaene et al., 1993). Alternatively, the SNARC effect has been proposed to result from a temporary association of numbers and space to be formed in WM, rather than reflecting a long-term memory representation along a mental number line (Herrera et al., 2008; van Dijck et al., 2009; Fias et al., 2011; Ginsburg et al., 2014). Accordingly, digits would be stored in WM in their canonical order during numerical tasks, with positions from the beginning/end of the sequence eliciting faster left-/right-sided responses respectively, thereby giving rise to the SNARC effect. Evidence in favor of the latter account was provided by studies showing that the SNARC effect indeed critically depended on the availability of WM resources (Herrera et al., 2008; van Dijck et al., 2009). Regardless of which theory might prevail, the SNARC effect is affected by great inter-individual variability, which depends amongst others on arithmetic performance (Hoffmann et al., 2014a, but see Cipora and Nuerk, 2013), spatial skills (Viarouge et al., 2014), and inhibitory control (Hoffmann et al., 2014b).

In the present study, we investigated whether math anxiety depends on the strength of number–space associations in the classical parity judgment task (i.e., parity SNARC effect) in university students. Moreover, we assessed the symbolic NDE, basic spatial skills, arithmetic performance, visuospatial and verbal WM, and inhibitory control. Apart from complementing previous observations about the link between math anxiety, arithmetic performance, and executive control as well as extending recent evidence about its association with basic numerical and spatial skills, the study outcomes should reveal for the first time whether math anxiety also relates to the spatial nature of numerical representations. This will shed further light onto the particular characteristics of basic number processing potentially constituting a risk factor of math anxiety. Since stronger SNARC effects were shown to be associated with stronger NDE (Viarouge et al., 2014), lower spatial skills (Viarouge et al., 2014), worse arithmetic performance (Hoffmann et al., 2014a), and weaker inhibitory control (Hoffmann et al., 2014b), which all relate to greater math anxiety, we hypothesized that individuals with HMA should display stronger number–space associations than their LMA peers.

## Materials and Methods

### Participants

A total of 86 students participated in this study, gave written informed consent and received 30€ for their participation. The study was approved by the Ethics Review Panel (ERP) of the University of Luxembourg. All students were recruited via advertisement through their university e-mail addresses. Since the present experiments were conducted in the context of a larger study examining amongst others the effects of mathematical expertise on number–space associations, students were recruited from different mathematical backgrounds. Half of the students came from study fields with a clear absence of explicit daily number and mathematics use (e.g., social and language studies), while the remaining participants all studied math-related subjects (e.g., mathematics, economics, or engineering). Recruitment within the two different math expertise levels was gender-balanced. Mathematical expertise was, however, not included as a between-subject factor, since it was not part of the aim of the current analyses.

Three participants had to be excluded from the sample due to a diagnosis of either attention-deficit/hyperactivity disorder (ADHD) or dyslexia. None of the 83 remaining participants reported to have any math-related or other learning difficulties and/or neuropsychological disorders. After exclusion of the three participants, outliers were identified for each of the different measures included in this study. A total of 18 participants were removed from the population sample, since their performances fell 2.5 standard deviations (SDs) below or above the mean group performances on at least one of these measures. Moreover, two participants were excluded due to a misinterpretation of task instructions. More details on outlier removal can be found in the Supplementary Material. The 63 remaining participants were assigned to either a low (LMA) or a high (HMA) math anxiety group based on a median-split procedure (Young et al., 2012; Rubinsten et al., 2015). Participants featuring overall math anxiety scores below or above the population median score (Median = 50) constituted the former or latter groups respectively. Two participants with math anxiety scores equal to the median value were excluded from analyses. The final sample thus consisted of 61 participants, including 31 LMA and 30 HMA individuals.

### Procedure and Tasks

The study comprised 12 tests consisting of questionnaires, paper-and-pencil exercises and computerized tasks. All computerized tasks were programmed in E-prime (Version 1.2 or 2.0.8.79) and administered using a Dell Laptop with a 15.6 in. color monitor (1024 × 768 Pixels).

Participants were tested individually during two 90 min testing sessions. Sessions were run on separate days to prevent any possible effects of fatigue. The time difference between the two testing sessions was not fixed, so that students could sign up for the sessions according to their preferences (e.g., during their free-time on campus between two lectures). The upper limit of 1 week between testing sessions was implemented to avoid too much variability in the range of time differences between sessions across participants. Time differences between sessions ranged from 1 day to 1 week in both math anxiety groups.

Considering that we performed correlation and regression analyses, all participants performed the tests in the same fixed order as indicated in **Table 1**. According to Carlson and Moses (2001), a fixed order is standard practice and advisable in individual differences research, since interpreting correlations from designs in which order has been counterbalanced might be hazardous.^1^ In addition to the fixed order of the tests, trial sequences were identical for all participants in every task. However, they were pseudo-randomized in a way that the correct response could not be on the same side more than two or three times consecutively in all the binary classification tasks.

**Table 1:** Order of the tests and the cognitive variables they assess on testing days one and two.

Order	Testing day one		Testing day two	
	Test	Cognitive variable	Test	Cognitive variable
1	OSIQ	Spatial visualization	Incompatibility task	Inhibitory control (IES difference)
2	Speeded matching to sample task	General processing speed	No grid WM task	Visuospatial WM
3	Parity judgment task	Parity SNARC effect	Categories subtest of the SON-R 6-40	Reasoning ability
4	Mental rotations test	Mental rotation	Untimed battery of arithmetic operations	Arithmetic performance (ArithACC)
5	Digit span subtest	Verbal WM	aMARS	Math anxiety
6	Magnitude comparison task	Distance effect		
7	FastMath task	Arithmetic performance (FastMathACC; FastMathRT)		

*OSIQ, Object spatial imagery questionnaire; SON-R 6-40, revised Snijders-Oomen non-verbal intelligence test 6-40; aMARS, abbreviated math anxiety rating scale questionnaire.*

#### Abbreviated Math Anxiety Rating Scale

*Math anxiety* was assessed using the abbreviated math anxiety rating scale (aMARS; Alexander and Martray, 1989; Baloǧlu and Zelhart, 2007), comprising 25 items. Participants were instructed to report their level of anxiety for each item on a five-point Likert-scale, with 1 for “not at all anxious” and 5 for “very much anxious.” The math anxiety score for each participant was calculated as the sum of all 25 item-scores. Individual levels of math anxiety could thus range from 25 to 125, with increasing scores reflecting an increased level of anxiety.

#### Parity Judgment and Magnitude Comparison Tasks

*Number–space associations* (SNARC effect) and the *numerical distance effect* (NDE) were calculated in the parity judgment and magnitude comparison tasks respectively.

The design of the parity judgment task was adapted from Dehaene et al. (1993) and is described in more detail in the Supplementary Material. On each trial, one of eight possible stimuli (1, 2, 3, 4, 6, 7, 8, or 9) appeared centrally. In the first block, participants judged as quickly as possible whether it was odd/even by pressing the “A”/“L” key on a QWERTZ keyboard respectively. This stimulus-response mapping was reversed for all participants in the second block. Each digit was displayed nine times per block. Each block started with 12–20 training trials, depending on response accuracy.

The design of the magnitude comparison task was adapted from van Galen and Reitsma (2008). The experiment was identical to the parity judgment task with the exception that participants judged whether the centrally presented digit was smaller/larger than five by pressing the “A”/“L” key respectively in the first block. This stimulus-response mapping was reversed for all participants in the second block.

Data from the training sessions was not analyzed. The mean error rate on experimental trials was 2.7 and 1.96% in the parity judgment and magnitude comparison tasks respectively. Errors were not further analyzed. Reaction times (RTs) shorter or longer than 2.5 SDs from the individual mean were considered outliers and discarded prior to data analysis (3.03 and 3% of all correct trials in the parity judgment and magnitude comparison tasks respectively). The SNARC effect and the NDE were determined using both the individual regression equations method (Fias et al., 1996) and the repeated measures ANOVA and linear trends method (Pinhas et al., 2012).

The individual regression equations method provides a single numerical value for both the SNARC effect and the NDE for every participant. To determine the SNARC effect, RTs were averaged separately for each digit and each response side (left/right) for every participant. Individual RT differences (dRTs) were then calculated by subtracting for each digit the mean left-sided RT from the mean right-sided RT. The resulting dRTs were subsequently submitted to a regression analysis, using the magnitude of individual digits as predictor variable. To calculate the NDE, trials were grouped based on the absolute value of the distance to the reference digit 5. Mean RTs were then calculated for each of the four distances (1, 2, 3, or 4) and regressed onto numerical distance for every participant. Unstandardized regression slopes were taken as a measure for both effects. Negative regression slopes indicated a SNARC effect in the expected direction (faster left/right-sided responses for small/large digits respectively) and the presence of a NDE. More negative regression slopes corresponded to stronger effects. To determine whether the SNARC effect and the NDE were significant at the group level, unstandardized regression slopes were tested against zero using a one-sample *t*-test.

The repeated measures ANOVA and linear trends method was used to determine the SNARC effect and NDE at the group level. To calculate the SNARC effect, an ANOVA was performed on mean dRTs including magnitude as within-subject variable. However, to avoid biases induced by possible MARC (Linguistic Markedness of Response Codes) effects (left-/right-sided advantages for odd/even digits respectively; Nuerk et al., 2004), RTs were collapsed to an even and an odd digit separately for each response side and each participant (as suggested by Pinhas et al., 2012; Tzelgov et al., 2013) and dRTs were computed for each of the four resulting magnitude categories (i.e., very small [1, 2], small [3, 4], large [6, 7], and very large [8, 9]). To determine the NDE, an ANOVA was conducted on RTs including numerical distance as a within-subject factor. SNARC effect and NDE were revealed by a significant main effect of magnitude and numerical distance respectively associated with a significant linear trend. Effect sizes of the linear trends provided information about the strengths of the effects.

Split-half reliabilities were calculated for the SNARC and NDE regression slopes using the odd–even method to control for systematic influences of practice or tiring within the tasks. Trials were odd–even half-split (based on order of appearance) and two regression slopes were calculated separately for each effect in every participant. The correlation coefficients were Spearman–Brown corrected to get a reliability estimate for the entire set of items. Reliabilities (SNARC effect: *r* = 0.58; NDE: *r* = 0.5) were sufficiently high to allow for subsequent interpretation of correlation and regression outcomes.

#### Mental Rotations Test and Object Spatial Imagery Questionnaire

*Mental rotation ability* was assessed using the 24-item MRT-A by Peters et al. (1995). For each item, participants were presented with a target figure and four comparison figures, of which two were rotated versions and two were mirror images of the target figure. Participants had 8 min to identify the two rotated versions of each target figure. Mental rotation ability (MRscore) was given by the number of items where both rotated versions of the target figure were correctly identified (i.e., maximum score = 24).

*Spatial visualization style* was determined using the object spatial imagery questionnaire (OSIQ) by Blajenkova et al. (2006). This is a 30-item questionnaire consisting of 15 spatial scale items and 15 object scale items, assessing spatial visualization and object visualization respectively. Participants were asked to rate each of the items on a five-point scale with 1 labeled ‘totally disagree’ and 5 labeled ‘totally agree.’ Since we did not have any specific hypotheses regarding the participants’ object visualization style, we only computed average scores for the spatial scale items for every participant (SVscore).

Similar to Kozhevnikov et al. (2010), scores from both tasks were normalized within the population and a composite score was computed as follows: zSpatial = zMRscore + zSVscore. This composite score provided us with a single measure of each participant’s spatial skills and was used for correlation analyses.

#### Untimed Battery of Arithmetic Operations and Timed FastMath Task

*Arithmetic performance* was assessed using the untimed battery of arithmetic operations (Shalev et al., 2001; Rubinsten and Henik, 2005), consisting of 20 number facts, 32 complex arithmetic problems, eight decimal problems, and 20 fractions. As in Hoffmann et al. (2014a), we scored 1 point for every correctly solved arithmetic problem and expressed accuracy as a percentage (ArithACC). We also administered the timed computerized FastMath task described in detail by Mussolin et al. (2012; see also Hoffmann et al., 2014a). The task consisted of 20 additions, multiplications, and subtractions on one- or two-digit Arabic numbers. All participants started with additions and finished with subtractions. We computed the accuracy (expressed as a percentage; FastMathACC) and the mean RT of all correct trials (FastMathRT) for each participant.

To compare our data to Hoffmann et al. (2014a), accuracy scores from both tasks and RTs were normalized within the population and a composite score was computed as follows: zArithmetic = zArithACC + zFastMathACC – zFastMathRT. This composite score provided us with a single measure of each participant’s arithmetic performance and was used for correlation analyses.

#### No Grid Visuospatial WM Task

*Visuospatial WM* was assessed using the grid/no grid WM task developed and described in detail by Martin et al. (2008). Participants had to remember the spatial locations of black target crosses, sequentially displayed in a 4 × 4 pattern. In contrast to Martin et al. (2008), only the no grid protocol was implemented, where the 16 possible spatial locations of the target crosses were not explicitly outlined by a grid. At the end of each trial, a comparison figure appeared, consisting of a configuration of darkened squares in a 4 × 4 subdivision of the background. Participants had to press the “A”/“L” key on a QWERTZ keyboard if the comparison configuration was in accordance/not in accordance with the spatial locations of the target crosses. WM load increased progressively over 36 trials from three to five target crosses. d prime (d^′^) was used as an index of visuospatial WM and computed for every participant by subtracting the false alarm rate (i.e., the proportion of incorrect responses on “no correspondence” trials) from the hit rate (i.e., the proportion of correct responses on “correspondence” trials).

#### Digit Span Subtest of the WAIS-III Battery

*Verbal WM* was assessed using the digit span subtest of the WAIS-III battery (Wechsler, 1997). We only administered the backward digit span version. Participants’ backward digit span was given by the number of correctly recalled sequences.

#### Incompatibility Task

*Inhibitory control* was assessed using a self-designed incompatibility task described in more detail in the Supplementary Material. The task consisted of experimental and catch trials. On experimental trials, a horizontal arrow was presented centrally in green/red on a 50/50 basis and pointed to the left/right on half of the trials. Participants had to judge the color of the arrow by pressing the “A”/“L” key on a QWERTZ keyboard for green/red arrows respectively regardless of the pointing direction. If the pointing direction of the arrow and the correct response side were the same/opposed, trials were considered as compatible/incompatible respectively. Catch trials were identical to experimental trials except that a green/red rhombus was displayed centrally instead of the arrow. Participants were instructed not to give a response. Catch trials were included to ensure that participants processed the irrelevant spatial dimension of the arrows before making a response based on their color. Individual error rates were determined for each compatibility condition on experimental trials and on catch trials. Individual mean correct RTs were calculated on compatible and incompatible trials after excluding outliers falling 2.5 SDs from the individual means.

#### Speeded Matching to Sample Task

*General processing speed* was determined using the speeded matching to sample task described in detail by Hoffmann et al. (2014a). Each trial consisted of a centrally displayed target shape and two possible solution shapes, displayed below to the left and right. Participants had to identify the solution that was identical to the target as quickly as possible by clicking the “A”/“L” key on a QWERTZ keyboard if it appeared on the bottom left/right respectively. General processing speed was determined by averaging RTs across all correct trials.

#### Revised Snijders-Oomen Non-verbal Intelligence Test 6-40

*Reasoning ability* was ascertained using the categories subtest of the revised Snijders-Oomen non-verbal intelligence test 6-40 (SON-R 6-40). Each of the 36 items consisted of three target pictures all belonging to a certain category and five option pictures of which two possessed the same categorical features than the target pictures. Participants were instructed to point toward the two option pictures that they would associate with the target ones. Items were scored as correct only if both of the option pictures were correctly identified, yielding a maximum score of 36.

## Results

### Group Comparisons

According to a Chi-square test of independence, math anxiety groups did not differ in terms of gender [*X*^2^(1) = 0.14; *p* = 0.71]. A one-way ANOVA on math anxiety scores (*M* = 54.66; *SD* = 20.0; ranging from 26 to 104) including gender as a between-subject variable did not reveal a main effect [*F*(1,59) = 0.29; *p* = 0.59; $\eta_{p}^{2}$ = 0.01], confirming similar levels of math anxiety across women and men. Furthermore, LMA and HMA individuals did not differ in age [*F*(1,59) = 0.001; *p* = 0.98; $\eta_{p}^{2}$ = 0.0]. All descriptive information for the two math anxiety groups can be found in **Table 2**.

**Table 2:** Descriptive information for the low and high math anxiety groups.

Variable	Math anxiety group	
	Low	High
Gender (f/m)	13/18	14/16
Age (years)	23.3 (3.34)	23.28 (3.02)
Handedness (r/l)	30/1	29/1
Math anxiety (score)	38.19 (6.51)	71.67 (13.95)
Parity SNARC effect (slope)	-6.43 (8.64)	-16.84 (14.52)
Distance effect (slope)	-10.29 (6.83)	-15.06 (11.1)
Mental rotation (score)	14.45 (5.38)	12.7 (5.05)
Spatial visualization (score)	3.05 (0.63)	2.93 (0.65)
ArithACC (%)	93.39 (4.38)	91.17 (6.08)
FastMathACC (%)	92.93 (4.37)	92.47 (5.15)
FastMathRT (ms)	2326 (810)	2687 (1027)
Visuospatial WM (d^′^)	0.77 (0.15)	0.66 (0.16)
Verbal WM (backward digit span)	7.13 (1.48)	7.07 (1.84)
Compatible IES (ms)	460 (62)	502 (74)
Incompatible IES (ms)	543 (74)	625 (93)
General processing speed (ms)	466 (79)	500 (119)
Reasoning ability (score)	27.1 (4.61)	26 (4.63)

*Standard deviations are shown in parentheses.*

#### Basic Numerical Processing

**FIGURE 1:**
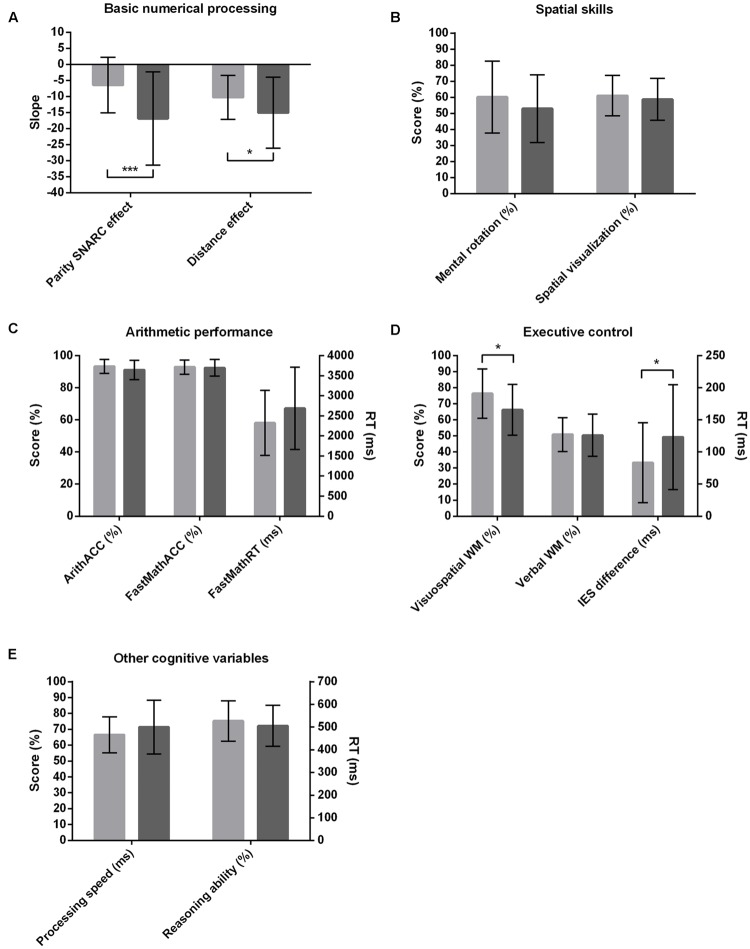
**Performances in the low (bright shading) and high (dark shading) math anxiety groups in the tasks probing basic numerical processing (A), spatial skills (B), arithmetic performance (C), executive control (D), and cognitive variables such as processing speed and reasoning ability (E).** Score, d^′^ and span values are expressed as percentages of maximum values. Error bars represent standard deviations. ^∗^*p* < 0.05; ^∗∗∗^*p* < 0.001.

The mean parity SNARC regression slope across all participants was -11.55 (*SD* = 12.91) and significantly differed from zero [*t*(60) = -6.99; *p* < 0.001], revealing a significant number–space association at the population level. A two-way ANOVA on the parity SNARC regression slopes including math anxiety group and gender as between-subject variables revealed a main effect of math anxiety group [*F*(1,57) = 11.48; *p* < 0.001; $\eta_{p}^{2}$ = 0.17], with HMA individuals featuring a significantly stronger parity SNARC effect than their LMA peers (HMA: slope = -16.84; *SD* = 14.52 vs. LMA: slope = -6.43; *SD* = 8.64; see **Figure 1A**). There was no effect of gender and no significant interaction between gender and math anxiety group. A two-way repeated measures ANOVA on mean parity dRTs including magnitude category (very small, small, large, very large) as within-subject variable and math anxiety group and gender as between-subject variables revealed a main effect of magnitude category [*F*(3,171) = 27.05; *p* < 0.001; $\eta_{p}^{2}$ = 0.32] associated with a significant linear trend [*F*(1,57) = 56.95; *p* < 0.001; $\eta_{p}^{2}$ = 0.5], thereby confirming the significant number–space association at the population level. However, most importantly and also in accordance with the aforementioned regression slope analysis, a significant interaction was found between magnitude category and math anxiety group [*F*(3,171) = 6.41; *p* < 0.001; $\eta_{p}^{2}$ = 0.1]. In both groups, main effects of magnitude category with associated linear trends were observed [HMA: main effect of magnitude category *F*(3,87) = 21.56; *p* < 0.001; $\eta_{p}^{2}$ = 0.43; associated linear trend *F*(1,29) = 43.48; *p* < 0.001; $\eta_{p}^{2}$ = 0.6 vs. LMA: main effect of magnitude category *F*(3,90) = 8.64; *p* < 0.001; $\eta_{p}^{2}$ = 0.22; associated linear trend *F*(1,30) = 16.25; *p* < 0.001; $\eta_{p}^{2}$ = 0.35]. HMA individuals, however, featured stronger number–space associations, as indicated by their greater effect size (HMA: $\eta_{p}^{2}$ = 0.43 vs. LMA: $\eta_{p}^{2}$ = 0.22). As for the regression slope analysis, no other effects and/or interactions reached significance.

The mean NDE regression slope across all participants was -12.64 (*SD* = 9.42) and significantly differed from zero [*t*(60) = -10.48; *p* < 0.001], indicating a significant distance effect at the population level. A two-way ANOVA on NDE regression slopes including math anxiety group and gender as between-subject variables revealed a main effect of math anxiety group [*F*(1,57) = 4.66; *p* = 0.04; $\eta_{p}^{2}$ = 0.08], with HMA individuals featuring significantly stronger distance effects than their LMA peers (HMA: slope = -15.06; *SD* = 11.1 vs. LMA: slope = -10.29; *SD* = 6.83; see **Figure 1A**).There was no effect of gender and no significant interaction. A two-way repeated measures ANOVA on mean RTs including distance as a within-subject factor and math anxiety group and gender as between-subject variables confirmed a main effect of distance [*F*(3,171) = 40.72; *p* < 0.001; $\eta_{p}^{2}$ = 0.42] associated with a significant linear trend [*F*(1,57) = 114.26; *p* < 0.001; $\eta_{p}^{2}$ = 0.67], again highlighting the presence of a distance effect at the population level. Moreover, analysis revealed a main effect of math anxiety group [*F*(1,57) = 4.4; *p* = 0.04; $\eta_{p}^{2}$ = 0.07], with LMA individuals responding on average faster than their HMA peers regardless of distance (LMA: RT = 479 ms; *SD* = 67 ms vs. HMA: RT = 510 ms; *SD* = 65 ms). However, contrary to the regression slope analysis, the interaction between math anxiety group and distance did not reach significance [*F*(3,171) = 2.14; *p* = 0.1; $\eta_{p}^{2}$ = 0.04]. There was no main effect of gender and no significant interactions.

#### Spatial Skills

The mean MRscore across all participants was 13.59 (*SD* = 5.25; ranging from 3 to 23). A two-way ANOVA on MRscore including math anxiety group and gender as between-subject variables revealed a main effect of gender [*F*(1,57) = 4.81; *p* = 0.03; $\eta_{p}^{2}$ = 0.08], with men reaching a significantly higher score than women (male MRscore = 14.88; *SD* = 4.91 vs. female MRscore = 11.96; *SD* = 5.31). There was no main effect of math anxiety group and no interaction (see **Figure 1B**).

The mean SVscore across all participants was 3 (*SD* = 0.64; ranging from 1.47 to 4.27). A two-way ANOVA on SVscore including math anxiety group and gender as between-subject variables did not reveal any main effects or interactions (see **Figure 1B**), indicating that groups did not differ in terms of their spatial visualization styles.

#### Arithmetic Performance

Mean ArithACC and FastMathACC across all participants were 92.3% (*SD* = 5.36; ranging from 78.75 to 100%) and 92.7% (*SD* = 4.74; ranging from 78.33 to 99.17%) respectively. Mean FastMathRT was 2504 ms (*SD* = 934 ms; ranging from 976 to 5322 ms). Three separate two-way ANOVAs on either ArithACC, FastMathACC, or FastMathRT including math anxiety group and gender as between-subject variables did not reveal any main effects or interactions (see **Figure 1C**). The different groups did thus not differ in terms of their arithmetic performance.

#### Working Memory

The mean visuospatial d^′^ value across all participants was 0.71 (*SD* = 0.16; ranging from 0.33 to 1). A two-way ANOVA on d^′^ values including math anxiety group and gender as between-subject variables revealed a main effect of math anxiety group [*F*(1,57) = 6.2; *p* = 0.02; $\eta_{p}^{2}$ = 0.1; HMA: *d^′^* = 0.66; *SD* = 0.16 vs. LMA: *d^′^* = 0.77; *SD* = 0.15; see **Figure 1D**], but no effect of gender or interaction. Results thus suggest that HMA individuals featured significantly worse visuospatial WM than their LMA peers regardless of gender.

The mean backward digit span across all participants was 7.1 (*SD* = 1.65; ranging from 4 to 11). A two-way ANOVA on digit span including math anxiety group and gender as between-subject variables did not reveal any main effects or interaction (see **Figure 1D**).

#### Inhibitory Control

The relatively low overall error rate on catch trials (6.35%; *SD* = 13.23%) suggested that participants attended to the spatial dimension of the target stimuli. A two-way ANOVA on error rates did not reveal any main effects of math anxiety group or gender nor a significant interaction.

The mean error rates and RTs across all participants on experimental trials were 1.23% (*SD* = 2.51%) and 474 ms (*SD* = 65 ms) in the compatible and 6.76% (*SD* = 6.87%) and 540 ms (*SD* = 64 ms) in the incompatible conditions respectively. Error rates and RTs correlated only in the compatible condition (compatible condition: *r* = 0.28; *p* = 0.03; incompatible condition: *r* = 0.19; *p* = 0.15), suggesting that these performance estimates partly provide different aspects of inhibitory control and that both measures need to be retained for further analyses. To incorporate the two variables into a single performance measure, we computed inverse efficiency scores (IES) by dividing the means of either compatible or incompatible correct RTs by their corresponding percentage accuracies for each participant (Bruyer and Brysbaert, 2011; Khng and Lee, 2014). IES thus adjusts RT performance for sacrifices in accuracy made in favor of response speed. Considering that faster responses together with fewer errors yield smaller IES, the smaller the IES is, the better the performance is.

A repeated measures ANOVA on IES including compatibility condition as within-subject variable revealed a main effect [*F*(1,60) = 116.41; *p* < 0.001; $\eta_{p}^{2}$ = 0.66], highlighting worse performance on incompatible (IES = 583.6 ms; *SD* = 93.03 ms) than compatible (IES = 480.52 ms; *SD* = 71.02 ms) trials at the population level. To get a single inhibitory control measure for each participant, we calculated IES differences by subtracting compatible from incompatible IES. A greater IES difference is indicative of weaker inhibitory control, as it reflects considerably worse performance (i.e., slower RT and/or more errors) on the incompatible compared to the compatible condition. A two-way ANOVA on IES differences including math anxiety group and gender as between-subject variables revealed a main effect of math anxiety group [*F*(1,57) = 4.21; *p* = 0.05; $\eta_{p}^{2}$ = 0.07], with HMA individuals featuring greater IES differences and thus weaker inhibitory control than their LMA peers (HMA: IES difference = 123 ms; *SD* = 82 ms vs. LMA: IES difference = 83 ms; *SD* = 62 ms; see **Figure 1D**). There was no effect of gender and no interaction. IES differences were also used for the subsequent correlation analyses.

#### Other Cognitive Variables

The mean general processing speed and reasoning ability across all participants were 483 ms (*SD* = 101 ms; ranging from 343 to 861) and 26.56 (*SD* = 4.61; ranging from 14 to 35) respectively. As indicated by two separate two-way ANOVAs, none of these variables differed between HMA and LMA individuals or gender and there was no significant interaction between the independent factors (see **Figure 1E**). These estimates were mainly included to rule out any differences in general cognitive abilities between the math anxiety groups. Since groups did not differ in these measures and considering that we did not have any specific hypotheses regarding their effects on math anxiety scores, these factors were not considered in the subsequent correlation analyses.

### Correlation Analysis

Despite the tendency of previous studies in the field of math anxiety to run median-splits and divide participants into two (or more) groups based on their math anxiety scores (see, e.g., Hopko et al., 1998), we also included the continuous version of this variable for correlation analyses. Considering that performing median-splits is associated with disadvantages such as the loss of information and statistical power (Cohen, 1983), the arbitrary nature of the cut-offs, and the population-dependence of a participant’s group membership, additionally running correlation analyses provides us with a clearer and more complete picture of the study outcomes.

All correlation coefficients for *N* = 61 are displayed in **Table 3**. Similar results were obtained when including the two individuals with math anxiety scores equal to the median value of 50. Correlation coefficients for *N* = 63 can be found in the Supplementary Material.

**Table 3:** Correlation analysis for *N* = 61.

	Parity SNARC effect	Distance effect	zSpatial	zArithmetic	Visuospatial WM	Backward digit span	IES difference
Math anxiety score	**-0.41^∗∗^**	-0.31^∗^	-0.16	-0.25^∗^	-0.29^∗^	-0.05	0.24#
Parity SNARC effect		0.17	0.06	0.31^∗^	**0.42^∗∗^**	0.15	-0.24#
Distance effect			0.04	0.09	-0.11	-0.02	-0.05
zSpatial				**0.43^∗∗^**	0.26^∗^	0.02	-0.08
zArithmetic					0.34^∗∗^	0.18	0.06
Visuospatial WM						0.19	-0.07
Backward digit span							0.13

*^∗^p < 0.05; ^∗∗^p < 0.01; ^#^p < 0.07. Significant Holm***–***Bonferroni adjusted p***-***values are displayed in bold.*

**FIGURE 2:**
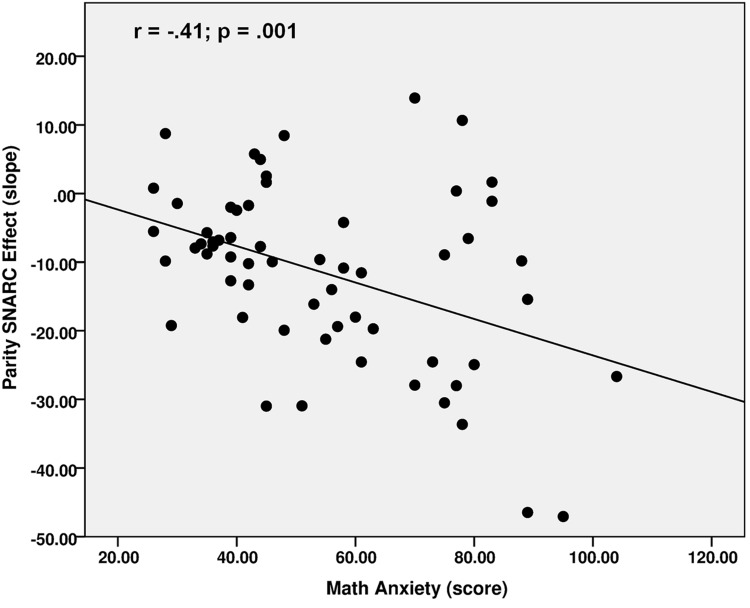
**Correlation between math anxiety scores and the parity SNARC effect regression slopes**.

A significantly negative correlation was observed between math anxiety scores and the parity SNARC regression slopes (*r* = -0.41; *p* = 0.001; see also **Figure 2**), highlighting greater math anxiety with stronger number–space associations during parity judgments. Math anxiety scores also correlated negatively with the NDE (*r* = -0.31; *p* = 0.02), indicating stronger distance effects in individuals with greater math anxiety. Conversely, no significant correlation was revealed between the math anxiety scores and zSpatial (*r* = -0.16; *p* = 0.21). This thus further confirms that the level of math anxiety is not related to spatial skills in our population. A significantly negative relationship was, however, revealed between math anxiety scores and zArithmetic (*r* = -0.25; *p* = 0.05), although group differences in arithmetic measures did not reach significance. Individuals with lower math anxiety scores thus featured better arithmetic performance. Math anxiety scores also negatively correlated with the d^′^ values of visuospatial WM (*r* = -0.29; *p* = 0.02). Conversely, backward digit spans were not related to math anxiety scores (*r* = -0.05; *p* = 0.69). These results thus indicate higher math anxiety with weaker visuospatial but not verbal WM. Finally, a positive trend could be observed between math anxiety scores and the IES difference (*r* = 0.24; *p* = 0.06). This supports the aforementioned significant group difference in this measure, highlighting weaker inhibitory control in individuals with HMA.

Interestingly, the parity SNARC effect and NDE were unrelated (*r* = 0.17; *p* = 0.19), suggesting that they reflect different properties of basic numerical processing. The parity SNARC effect, however, significantly correlated with Arithmetic (*r* = 0.31; *p* = 0.02), replicating previous observations about stronger number–space associations in individuals with weaker arithmetic performance (Hoffmann et al., 2014a). A significantly positive correlation was also observed between the parity SNARC effect and visuospatial d^′^ values (*r* = 0.42; *p* = 0.001), highlighting weaker number–space associations in individuals with better visuospatial WM. There was also a tendency for an association between the parity SNARC effect and IES difference (*r* = -0.24; *p* = 0.07), indicating stronger number–space associations with weaker inhibitory control (for similar results see Hoffmann et al., 2014b). The observation that visuospatial and verbal WMs were unrelated (*r* = 0.19; *p* = 0.15), indicated that they rely, at least partly, on different cognitive mechanisms. Moreover, visuospatial but not verbal WM correlated with zArithmetic (visuospatial: *r* = 0.34; *p* = 0.01 vs. verbal: *r* = 0.18; *p* = 0.17). zArithmetic was also significantly positively associated with zSpatial (*r* = 0.43; *p* = 0.001), which in turn correlated with visuospatial WM (*r* = 0.26; *p* = 0.05).

Including gender as a covariate in a partial correlation analysis did not change any of the aforementioned outcomes. All partial correlation coefficients for *N* = 61 can be found in the Supplementary Material.

Considering that we performed a large number of correlations, the Holm–Bonferroni method was applied to correct the results for multiple comparisons (Holm, 1979). Since this technique is more powerful than the classical Bonferroni method and maintains the overall rate of false positives without inflating the rate of false negatives unnecessarily, it was the procedure of choice for the present analyses. The relation between math anxiety and the parity SNARC effect remained significant even after applying the Holm–Bonferroni sequential correction (adjusted *p*-value = 0.03). Significant Holm–Bonferroni adjusted *p*-values are displayed in **Table 3**.

### Multiple Regression Analysis

Taking into account the recent findings and theories suggesting that deficits in basic numerical skills contribute to the development of math anxiety and also considering that the main aim of the present study was to determine whether number–space associations are another potential risk factor of math anxiety, we performed stepwise multiple linear regression analysis on math anxiety scores as the dependent variable. In addition to basic numerical skills (i.e., the parity SNARC effect and the NDE) zArithmetic, visuospatial WM and IES difference were included as predictors, since these variables are commonly associated with math anxiety and also correlated with the latter in the present study. This analysis should allow us to determine the best set of predictors of math anxiety. The results will especially inform us about the predictive power of basic numerical skills when controlling for the effects of arithmetic performance and executive control.

The prediction model contained two out of the five predictors and was reached in two steps with no variables removed. The model was statistically significant [*F*(2,58) = 8.51; *p* = 0.001] and accounted for approximately 23% of the variance of math anxiety scores (*r* = 0.48; *R*^2^ = 0.23; adjusted *R*^2^ = 0.2). The parity SNARC effect and the NDE were significant predictors of math anxiety scores, with the parity SNARC effect receiving the strongest weight in the model. The regression outcome thus suggests that math anxiety was primarily predicted by the strength of number–space associations in the parity judgment task and to a slightly lesser extent by the NDE. No additional variance could be explained by arithmetic performance, visuospatial WM or inhibitory control. Raw and standardized regression coefficients of the predictors are shown in **Table 4**.

**Table 4:** Stepwise multiple linear regression analysis on math anxiety scores.

Model	*B*	*SE-B*	β	*t*	*p*
Constant	41.49	4.16		9.98	<0.001
Parity SNARC effect	-0.57	0.18	-0.37	-3.16	0.003
Distance effect	-0.52	0.25	-0.24	-2.08	0.04

*R^2^ = 0.23; adj. R^2^ = 0.2; F(2,58) = 8.51; p = 0.001.*

## Discussion

Considering recent findings suggesting that deficits in basic numerical processing and spatial skills might be at the origin of math anxiety (Maloney et al., 2010, 2011, 2012; Núñez-Peña and Suárez-Pellicioni, 2014; Dietrich et al., 2015; Ferguson et al., 2015), the present study aimed to determine whether the quality of spatial-numerical associations might also be a potential risk factor of math anxiety. Furthermore, we aimed to replicate the relation between math anxiety and basic numerical and spatial skills, in addition to confirming its well-established associations with arithmetic performance, WM, and inhibitory control.

**FIGURE 3:**
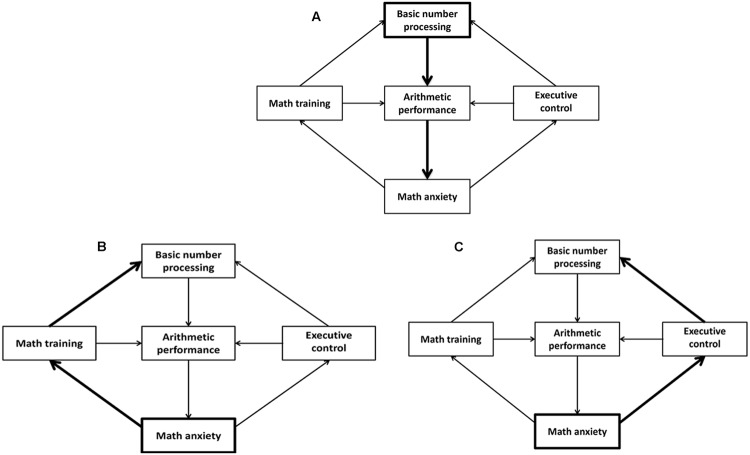
**Schematic representations of the different mechanisms potentially accounting for the relationship between math anxiety and basic numerical processing (SNARC effect)**. Deficits in basic numerical processing lead to math anxiety via weaker arithmetic performance **(A)**. Math anxiety leads to deficits in basic numerical processing via insufficient math training **(B)** or weaker executive control **(C)**. The origin and pathways for each of the different mechanisms are highlighted in bold.

As hypothesized, we found that greater math anxiety was associated with stronger spatio-numerical interactions in the parity judgment task. This novel finding thus strengthens the assumption that inadequacies in basic numerical abilities might be a potential risk factor of math anxiety. One possible explanation for this association might be that stronger reliance on concrete spatial aspects when dealing with abstract numerical information (as evidenced by stronger SNARC effects) might compromise the optimal development of higher-level mathematical competencies. This, in turn, might put individuals at risk of math failure, subsequently leading to the emergence of math anxiety (see **Figure 3A**). Of course, this theory relies on the assumption that the parity SNARC effect remains constant throughout development, such that the size of the SNARC effect assessed in university students can be used as an indicator of the strength of their number–space associations during the earlier years of mathematical learning. Support for the idea that stronger number–space associations might cause greater math anxiety via negatively impacting on mathematical performance is provided by recent observations, highlighting a link between stronger spatio-numerical interactions and lower proficiency in the application of basic math knowledge (Hoffmann et al., 2014a). Moreover, it is in line with a study on the causal order of math achievement and math anxiety, indicating that prior low math performance related to later HMA across junior and senior high school, but not vice versa (Ma and Xu, 2004).

In general, the idea that inadequate basic numerical skills, such as stronger SNARC effects, might constitute a risk factor for the emergence of math anxiety is in accordance with several observations in the field. For instance, Young et al. (2012) showed that greater math anxiety was associated with altered activity in the posterior parietal lobe already in children as young as first grade, which is a region involved in mathematical reasoning and also the presumed cognitive locus of the SNARC effect (Cutini et al., 2014). Furthermore, Maloney et al. (2011) reported that HMA individuals displayed stronger distance effects (see also Núñez-Peña and Suárez-Pellicioni, 2014; Dietrich et al., 2015, for similar results), which led the authors to suggest that a deficit in the representation of numerical magnitudes (i.e., a defective ANS) might be at the origin of math anxiety. Dietrich et al. (2015) only observed an association between math anxiety and the distance effect in a symbolic, but not in a non-symbolic dot comparison task. They therefore suggested that inadequate numerical comparison processes, rather than weaker ANS acuity (see also Van Opstal et al., 2008), might underlie the stronger distance effects in HMA individuals and constitute a risk factor of math anxiety. In line with these findings, Rubinsten and Tannock (2010) observed a strong relationship between developmental dyscalculia and math anxiety.

The link between the symbolic distance effect and math anxiety could also be replicated by the present study. Interestingly, however, we did not find a significant relationship between the NDE and the parity SNARC effect (see Herrera et al., 2008, for similar results; but also see Viarouge et al., 2014), assuming that both phenomena represent different basic numerical competencies whose functional weaknesses predispose individuals to the development of math anxiety. According to these findings, the nature of the numerical inadequacies ultimately leading to greater math anxiety seems to be multi-factorial and heterogeneous.

Although, the present study further confirmed the association between math anxiety and basic numerical skills, the recently observed relationship between math anxiety and basic spatial skills such as mental rotation ability and spatial visualization style could not be replicated (Maloney et al., 2012; Ferguson et al., 2015). Considering that the relationship between math anxiety and spatial abilities in the study of Ferguson et al. (2015) was shown to depend on spatial anxiety, differences in this factor and in its association with math anxiety and/or spatial skills in the present population might account for the discrepancy between current and previous findings. Moreover, the present sample was relatively small compared to that of Maloney et al. (2012) and Ferguson et al. (2015) and consisted only of highly educated university students with no general math difficulties or extreme levels of math anxiety. It is possible that a significant correlation between spatial skills and math anxiety might only be evidenced in a larger and broader population including individuals with more variable math anxiety scores and with spatial skills spanning the entire ability spectrum.

The present study could, however, confirm the well-documented negative relationship between math anxiety and arithmetic performance (Hembree, 1990; Ashcraft and Faust, 1994; Ma, 1999; Ashcraft and Kirk, 2001), at least when performing correlation analyses.

We were also able to replicate the association between math anxiety and WM (Ashcraft and Kirk, 2001; Ashcraft and Krause, 2007). The worrying intrusive thoughts associated with math anxiety are thought to consume the limited resources of WM, consequently leading to weaker performance on WM tasks. This has, amongst others, also been suggested as one of the mechanisms through which math anxiety negatively impacts on arithmetic performance (see competition for WM resources theory; Ashcraft and Faust, 1994; Ashcraft and Kirk, 2001; Ashcraft and Krause, 2007). An interesting point worth mentioning here is that the relation between math anxiety and WM could only be evidenced in the visuospatial but not the verbal task. This might seem surprising at first given the numerical content of the backward digit span test. Our results are, however, in accordance with previous findings, assessing the effect of math anxiety on the backward digit span in undergraduate students (Buelow and Frakey, 2013). Ashcraft and Kirk (2001) argued that WM might only be compromised in HMA individuals when the actual math anxiety is aroused, such as in a span task involving computations, since they only evidenced a WM decline in the latter situation. It might thus be possible that despite the numerical content of the backward digit span task, the lack of computations prevented the arousal of math anxiety, thereby explaining the absence of a performance drop in HMA individuals. Conversely, the visuospatial content of the no grid WM task might have been more reminiscent of a mathematical solution, and as such more likely to evoke feelings of anxiety, ultimately compromising WM performance. This might also explain why visuospatial but not verbal WM correlated with zArithmetic.

The link that we observed between math anxiety and inhibitory control also complements previous findings in the literature. For instance, in a numerical Stroop task, HMA individuals needed more time to state the quantity of numerical than non-numerical stimuli, while no difference in RTs could be observed for the LMA group (Hopko et al., 2002). HMA individuals thus seemed to have particular difficulties to focus on task-relevant information in interfering situations. In a similar vein, Pletzer et al. (2015) showed that the compatibility effect in a number comparison task was accompanied by higher neural activity in inhibitory control areas such as the inferior frontal cortex on incompatible trials for LMA but not HMA individuals, again suggesting an inhibitory deficit in the latter group. Finally, in a task where individuals were required to respond to the digits with greater numerical magnitude while ignoring their irrelevant physical size, Suárez-Pellicioni et al. (2014) found a greater degree of interference for RTs in the HMA than the LMA group. Hopko et al. (1998) suggested that the greater susceptibility to distraction among HMA individuals and their failure to inhibit attention to the worrying intrusive thoughts associated with math anxiety might be the actual cause underlying their depleted WM and the resulting performance deficits (see deficient attentional control theory).

A final point worth addressing here is that we did not find any gender differences in the level of math anxiety. In addition to this, gender did not interact with math anxiety group nor did it affect correlation outcomes when added as a covariate. While this is in accordance with a number of previous observations (e.g., Haynes et al., 2004; Birgin et al., 2010), it conflicts with other studies, reporting greater math anxiety in women (e.g., Hembree, 1990; Frenzel et al., 2007; Goetz et al., 2013). Gender differences are usually assumed to be driven by confounding factors such as the attitude toward mathematics rather than gender *per se* (Ashcraft and Ridley, 2005; Beilock et al., 2007). This might be one of the reasons for the absence of gender differences in the present population.

### Limitations and Outlook

Even though our main hypothesis was based on recent findings and theories suggesting that deficits in basic numerical and spatial skills were at the origin of math anxiety, our correlation and regression results cannot imply a causal relationship and as such it remains unclear whether stronger number–space associations are the causes or rather consequences of HMA. To shed further light onto this, one might for instance determine the effects of experimentally induced math anxiety (e.g., by exposing women to a stereotyping message regarding better math performance in men) on the strength of the parity SNARC effect.

Although, the idea that stronger number–space associations represent a risk factor of math anxiety finds abundant support in the current literature, a reverse association is also easily justifiable. For instance, the decline in math practice often associated with HMA (see global avoidance theory, Ashcraft and Faust, 1994) might entail greater reliance on concrete spatial aspects when dealing with abstract numerical concepts, thereby manifesting in stronger SNARC effects (see **Figure 3B**). Less trained individuals were indeed shown to have stronger number–space associations than professional mathematicians (Hoffmann et al., 2014a; Cipora et al., 2015). Alternatively, the greater susceptibility to distraction in HMA individuals (Hopko et al., 2002; Suárez-Pellicioni et al., 2014; Pletzer et al., 2015) might lead to greater interference of the irrelevant magnitude-associated spatial code during parity judgments, thereby resulting in stronger parity SNARC effects (see **Figure 3C**). Again, support for this idea is provided by Hoffmann et al. (2014b), reporting stronger number–space associations with weaker inhibitory control. To demonstrate the validity of the two aforementioned theories, one needs to assess whether math practice and/or inhibitory control actually mediate the relationship between math anxiety and spatio-numerical interactions.

Moreover, future studies should investigate the influence of possible covariates in greater detail. Math practice and/or executive control might for instance be confounding variables in the relation between math anxiety and the SNARC effect, rather than playing a mediating role. Attitude toward mathematics, confidence, and stereotypes could also be considered as potential covariates (Devine et al., 2012).

Furthermore, an extreme group approach (Preacher, 2015) could determine whether spatial skills differ between LMA and HMA groups when including only the lower and upper extremes of the math anxiety distribution (for the implementation of such a design see, e.g., Maloney et al., 2010, 2011; Lyons and Beilock, 2011; Núñez-Peña and Suárez-Pellicioni, 2014).

Finally, since the present study only included highly educated university students with no math difficulties, it needs to be verified whether our main conclusions can hold in a broader and more variable study sample.

## Conclusion

Taken together, the present study showed that greater math anxiety was significantly associated with stronger spatio-numerical interactions in addition to more pronounced distance effects. Moreover, these basic numerical processing skills predicted math anxiety over and above arithmetic performance, WM, and inhibitory control. These findings significantly add to the recent evidence supporting a crucial link between math anxiety and basic numerical abilities and strengthen the idea that deficits in the latter might constitute a potential risk factor of math anxiety (Maloney et al., 2010, 2011; Núñez-Peña and Suárez-Pellicioni, 2014; Dietrich et al., 2015).

## Author Contributions

Conceived and designed the experiments: CG, DH, CS. Analyzed the data: CG. Wrote the paper: CG, DH, CS.

## Conflict of Interest Statement

The authors declare that the research was conducted in the absence of any commercial or financial relationships that could be construed as a potential conflict of interest.

## Footnotes

1 The task order chosen in the present study is justified as follows. The parity judgment task was administered before the magnitude comparison task to prevent the priming of numerical magnitudes prior to completion of the former task. Arithmetic tasks were run on separate days to avoid overstraining participants especially those with high math anxiety. The math anxiety questionnaire was administered last to prevent the potential emphasis of the participants’ math anxiety through completion of this questionnaire from interfering with their performances specifically on numerical tasks.
