# Supplementary material for: How Math Anxiety Relates to Number–Space Associations
Source: Front Psychol. 2016 Sep 14;7:1401. doi: 10.3389/fpsyg.2016.01401 (PMC5021703; doi:10.3389/fpsyg.2016.01401)
Supplement: Supplementary file 1 [file Data_Sheet_1.DOCX]

Supplementary Material

How math anxiety relates to number-space associations

**Carrie Georges*, Danielle Hoffmann, Christine Schiltz**

*** Correspondence:** Carrie Georges: carrie.georges@uni.lu

# Supplementary methods

## Participant exclusion procedure

A total of 86 students participated in this study. Three participants were immediately excluded from analysis due to a diagnosis of either attention-deficit/hyperactivity disorder (ADHD) or dyslexia. After removal of those individuals, outliers were determined for each of the behavioural measures included in this study. If participants’ performances fell 2.5 SDs below or above the mean group performance on at least one of these measures, they were considered as outliers and excluded from all subsequent analyses. Since many different variables were analyzed for the incompatibility with catch task, we decided to ascertain outliers based on the participants’ overall performance on this task (i.e., average IES). A total of 18 participants appeared as outliers (some on more than one measure) and were excluded. The exact number of outliers removed for the different measures can be found in the Supplementary table 1 (see also Supplementary figure 1 for the visualization of outlier performances). In addition to these outliers, two participants were removed from all analyses since they misinterpreted the instructions on the speeded matching to sample task used to assess general processing speed. After the exclusion procedure, the study sample thus consisted of 63 participants. Participants were then assigned to either a low (LMA) or a high math anxiety (HMA) group based on a median-split procedure. Two individuals featured math anxiety scores equal to the median value and were excluded from all subsequent group, correlation and regression analyses. The final study sample therefore comprised 61 participants. Descriptive information for the different populations before and after the exclusion procedure can be found in the Supplementary table 1.

## Parity judgment task

The design of the parity judgment task was adapted from Dehaene et al. (1993). The experiment consisted of 144 experimental trials divided equally across two blocks. Each experimental trial started with an empty black-bordered square on a white background (sides 100 pixels, border 2 pixels). After 300 ms, one of eight possible stimuli (Arabic digits 1, 2, 3, 4, 6, 7, 8 or 9) presented in black on a white background in font Arial point size 64, appeared in the center of the black-bordered square and remained for 1300 ms. The inter-trial interval consisted of a blank screen of 1300 ms. Participants had to judge as quickly as possible whether the centrally presented single Arabic digit was odd or even by pressing either the ‘‘A’’ or ‘‘L’’ key on a standard QWERTZ keyboard. In the first block, all participants had to press the “A”/”L” key for odd/even digits respectively. This stimulus-response mapping was reversed for all participants in the second block. Each target digit was displayed exactly nine times per block. The sequence in which the target stimuli appeared was identical for all participants. However, it was pseudo-randomized in a way that no target digit could appear twice in a row, and the correct response could not be on the same side more than three times consecutively. Each block started with 12–20 training trials, depending on response accuracy. If accuracy was at least 70%, participants could directly proceed to the experimental trials after 12 training trials. If the threshold of 70% was not reached, another eight training trials were administered before the experimental trials started. Participants were given a small break half-way through each block.

## Incompatibility task

To assess inhibitory control, we administered a self-designed incompatibility task, consisting of 32 experimental trials and 8 catch trials. Experimental trials started with the presentation of a black fixation cross in font Courier New point size 18 in the center of a white background. After 400 ms, the fixation cross was replaced by a horizontal arrow that disappeared upon response or after a maximum of 1000 ms. The central arrow was presented in either green or red on a 50/50 basis. Regardless of its color, the arrow pointed to the left on half of the trials and to the right on the remaining half. Participants were instructed to judge the color of the arrow by pressing the “A”/“L” keys on a standard QWERTZ keyboard for green/red arrows respectively regardless of the pointing directions. If pointing direction coincided with correct response side, the experimental trials were considered as compatible. On incompatible trials, correct response side and the pointing direction of the arrow were opposed. The inter-trial interval consisted of a blank screen for 500 ms. Catch trials were identical to experimental trials with the exception that a rhombus was displayed in the center of the screen instead of the arrow. The rhombus appeared in either green or red, but regardless of its color, participants were instructed not to give a response. It disappeared in case of response or after a maximum of 1000 ms. Catch trials were included to ensure that participants processed the irrelevant spatial dimension of the arrows before making a response based on their color. Trial sequence was identical for all participants, but it was pseudo-randomized in a way that the correct response could not be the same more than 2 times consecutively. The actual experiment was preceded by 10 practice trials, consisting of 8 experimental trials and 2 catch trials.

# Supplementary results

## Correlation analyses

Correlation analyses were repeated with *N* = 63. Similar results were obtained regardless of whether the population sample consisted of 61 or 63 participants. Results are displayed in the Supplementary table 2.

Including gender as a covariate in a partial correlation analysis did not change any of the outcomes. All partial correlation coefficients for *N* = 61 can be found in the Supplementary table 3.

# Supplementary figures and tables

## Supplementary tables

**Supplementary table 1.** Descriptive information for the different study populations before and after the exclusion procedure and the number of outliers removed for each behavioural measure.

| Variable | **Study population** | | | ***N* of outliers** |
| --- | --- | --- | --- | --- |
|  | ***N* = 83** | ***N* = 63** | ***N* = 61** |  |
| Gender (f/m) | 40/43 | 29/34 | 27/34 | / |
| Age (years) | 23.45 (3.06) | 23.27 (3.12) | 23.29 (3.16) | / |
| Handedness (r/l) | 79/4 | 61/2 | 59/2 | / |
| Math anxiety (score) | 57.18 (20.08) | 54.51 (19.69) | 54.66 (20) | 0 |
| Parity SNARC effect (slope) | -11.34 (14.98) | -11.65 (12.74) | -11.55 (12.91) | 3 |
| Distance effect (slope) | -14.68 (14.24) | -12.73 (9.49) | -12.64 (9.42) | 3 |
| Mental rotation (score) | 13.08 (5.37) | 13.6 (5.2) | 13.59 (5.25) | 0 |
| Spatial visualization (score) | 2.98 (0.63) | 2.99 (0.63) | 2.99 (0.64) | 0 |
| ArithACC (%) | 90.81 (8.01) | 92.1 (5.45) | 92.3 (5.36) | 3 |
| FastMathACC (%) | 92.1 (6.8) | 92.57 (4.74) | 92.7 (4.74) | 1 |
| FastMathRT (ms) | 2660 (1128) | 2533 (973) | 2504 (934) | 2 |
| Visuospatial WM (d’) | .68 (.17) | .71 (.16) | .71 (.16) | 2 |
| Verbal WM (backward digit span) | 6.96 (1.65) | 7.08 (1.63) | 7.1 (1.65) | 0 |
| Average IES (ms) | 555 (115) | 533 (73) | 532 (74) | 2 |
| Compatible IES (ms) | 501 (111) | 481 (73) | 481 (71) | / |
| Incompatible IES (ms) | 609 (133) | 586 (94) | 584 (93) | / |
| General processing speed (ms) | 510 (144) | 482 (101) | 483 (101) | 3 |
| Reasoning ability (score) | 26.23 (5.07) | 26.37 (4.81) | 26.56 (4.61) | 1 |

Standard deviations are shown in parentheses.

**Supplementary table 2.** Correlation analysis for *N* = 63.

|  | **Parity SNARC effect** | **Distance effect** | **zSpatial** | **zArithmetic** | **Visuospatial WM** | **Backward digit span** | **IES difference** |
| --- | --- | --- | --- | --- | --- | --- | --- |
| **Math anxiety score** | -.41** | -.3* | -.16 | -.23# | -.29* | -.05 | .21 |
| **Parity SNARC effect** |  | .16 | .06 | .29* | .41** | .15 | -.24# |
| **Distance effect** |  |  | .05 | .13 | -.08 | -.03 | .05 |
| **zSpatial** |  |  |  | .42** | .26* | .02 | -.06 |
| **zArithmetic** |  |  |  |  | .36** | .18 | .09 |
| **Visuospatial WM** |  |  |  |  |  | .19 | -.03 |
| **Backward digit span** |  |  |  |  |  |  | .08 |

* *p* < .05; ** *p* < .01; *** *p* < .001; # p < .07.

## Supplementary table 3. Partial correlation analysis for *N* = 61 including gender as a covariate.

|  | **Parity SNARC effect** | **Distance effect** | **zSpatial** | **zArithmetic** | **Visuospatial WM** | **Backward digit span** | **IES difference** |
| --- | --- | --- | --- | --- | --- | --- | --- |
| **Math anxiety** | -.42** | -.3* | -.15 | -.25# | -.3* | -.06 | .25# |
| **Parity SNARC effect** |  | .17 | .08 | .31* | .42** | .14 | -.23# |
| **Distance effect** |  |  | .02 | .09 | -.11 | -.01 | -.05 |
| **zSpatial** |  |  |  | .44*** | .31* | .05 | -.1 |
| **zArithmetic** |  |  |  |  | .35** | .18 | .06 |
| **Visuospatial WM** |  |  |  |  |  | .18 | -.06 |
| **Backward digit span** |  |  |  |  |  |  | .13 |

* *p* < .05; ** *p* < .01; *** *p* < .001; # p < .07.

## Supplementary figure legend

## Supplementary figure 1. Visualization of outliers for the different measures. Score, d’ and span values are expressed as percentages of maximum values.
